# Description of STRIVE-ON Study Protocol: Safety and Tolerability of GTX-104 (Nimodipine Injection for IV Infusion) Compared with Oral Nimodipine in Patients Hospitalized for Aneurysmal Subarachnoid Hemorrhage (aSAH): A Prospective, Randomized, Phase III Trial (STRIVE-ON)

**DOI:** 10.1007/s12028-024-02207-8

**Published:** 2025-01-28

**Authors:** Alex H. Choi, Sherry Y. Chou, Andrew F. Ducruet, W. Taylor Kimberly, R. Loch Macdonald, Alejandro A. Rabinstein

**Affiliations:** 1 Division of Neurocritical Care, Department of Neurosurgery, UTHealth Houston, Houston, TX USA; 2 Division of Neurocritical Care, The Ken and Ruth Davee Department of Neurology, Northwestern Feinberg School of Medicine, Chicago, IL USA; 3 Neurosurgery, Barrow Neurological Institute, Phoenix, AZ USA; 4 Division of Neurocritical Care, Massachusetts General Hospital and Department of Neurology, Harvard Medical School, Boston, MA USA; 5 Community Regional Medical Center, Community Neurological Institute and Community Health Partners, Fresno, CA USA; 6 Department of Neurology, Mayo Clinic, Rochester, MN USA

**Keywords:** Calcium channel antagonist, Cerebral aneurysm, Cerebral vasospasm, Clinical trial, Delayed cerebral ischemia, Nimodipine, Subarachnoid hemorrhage

## Abstract

Oral nimodipine is the only drug approved in North America for patients with aneurysmal subarachnoid hemorrhage (aSAH). However, bioavailability is variable and frequently poor, leading to fluctuations in peak plasma concentrations that cause dose-limiting hypotension. Furthermore, administration is problematic in patients who cannot swallow. An oral liquid formulation exists but causes diarrhea. An intravenous nimodipine formulation (GTX-104) has been developed that has bioavailability approaching 100% and is not affected by feeding or gastrointestinal absorption. GTX-104 causes less hypotension and has more consistent peak plasma concentrations than oral nimodipine in human volunteers. Herein, we describe the protocol of a prospective, randomized, open-label safety, and tolerability study of GTX-104 compared with oral nimodipine in patients with aSAH (Safety and Tolerability of GTX-104 [Nimodipine Injection for Intravenous Infusion] Compared with Oral Nimodipine; ClinicalTrials.gov identifier: NCT05995405). The study is designed to seek approval of GTX-104 by the Food and Drug Administration 505(b)(2) pathway. Inclusion and exclusion criteria match the prescribing information for oral nimodipine and include adult patients with aSAH of all Hunt and Hess grades who can receive investigational product within 96 h of aSAH. Study participants at imminent risk of death will be excluded. Study participants will be randomly assigned 1:1 to receive GTX-104 or oral nimodipine for up to 21 days. The primary end point is the proportion of study participants with clinically significant hypotension, defined as hypotension requiring treatment that has a reasonable likelihood of being due to investigational product as determined by an independent, blinded end point adjudication committee. No statistical analysis of the end point is planned. Secondary end points include all episodes of hypotension, all adverse events, delayed cerebral ischemia, rescue therapy, and suicidal ideation. Clinical and health economic outcomes include quality of life using the EuroQol 5-dimension/3-level, modified Rankin Scale score at 30 and 90 days after aSAH and hospital resource use. The planned sample size is 100 study participants across 25 sites in the United States and Canada.

**Supplementary Information:**

The online version contains supplementary material available at 10.1007/s12028-024-02207-8.

## Introduction

This report adheres to the Standard Protocol Items: Recommended for Interventional Trials [1]. Nimodipine is the only drug approved for administration to patients with aneurysmal subarachnoid hemorrhage (aSAH) [2]. It is a dihydropyridine molecule that antagonizes L-type calcium channels that are located predominately on vascular smooth muscle. Two oral formulations (capsules, liquid solution) are available in the United States, whereas tablets and an intravenous (IV) formulation are available in the European Union and some other countries.

There are problems with the oral nimodipine capsules, including poor and variable bioavailability, high first-pass effect, risk of inadvertent IV injection, and need to aspirate the liquid from the capsules to administer to patients who cannot swallow if the oral liquid solution is not available [3, 4]. The oral nimodipine solution causes diarrhea [5]. These lead to poor compliance and unpredictable episodes of hypotension [6–10]. Hypotension remains the most important and frequent adverse effect of nimodipine [11]. The nimodipine prescribing information reports 5% of patients developed hypotension at the approved dosage. Another clinical trial reported oral nimodipine caused hypotension (defined as decrease in systolic blood pressure [BP] > 20 mm Hg, diastolic BP > 10 mm Hg, or systolic BP ≤ 90 mm Hg, confirmed by three consecutive readings and requiring medical treatment) in 10% of patients [12]. The frequency of dose reductions and discontinuations of oral nimodipine due to hypotension is even higher in observational studies and is reportedly as high as 44% [7, 9, 10, 13].

Although IV nimodipine might avoid some of these problems, there are limitations of the IV formulation of nimodipine. The safety and efficacy of nimodipine is based on the oral formulations. The IV solution has not been well studied in the nimodipine clinical trials, and a meta-analysis stated that there was insufficient data to define the benefits of IV nimodipine [14]. In addition to limited sample size, the IV nimodipine dose regimen is a continuous infusion and does not produce the saw-tooth pharmacokinetic (PK) profile of oral nimodipine. It is not known if the PK profile is important for the efficacy of nimodipine. Additionally, each 50-mL bottle of the IV formulation available in the European Union contains 10 g of ethanol (96%) and 10 mg of nimodipine. At the prescribed dosage, this equates to nearly a bottle of wine per day, which raises concern for liver toxicity. This formulation must be given through a central line because it causes vein irritation, pain, and inflammation [15]. Finally, hypotension is reported in approximately 30% of patients [6, 16–20].

A new formulation of IV nimodipine, GTX-104, could overcome the limitations of existing IV and oral nimodipine formulations. Potential advantages of GTX-104 include less interpatient and intrapatient variability in PK, less hypotension, almost 100% bioavailability because of no food effect or first-pass metabolism, and better compliance due to ease of administration to patients who cannot swallow.

### Phase I Clinical Data

GTX-104 has been administered to 104 healthy human volunteers in two studies (unpublished data, Grace Therapeutics). A phase I, single center, randomized, two-period crossover PK study assessed GTX-104 and oral nimodipine capsules, which are the reference standard, in 58 study participants.

GTX-104 was administered for 72 h as a continuous infusion of 0.15 mg/hour with a 30-min bolus infusion of 4 mg every 4 h. Nimodipine capsules were administered orally at a dosage of 60 mg (two 30-mg capsules) for 72 h. The two products demonstrated similar results for the two primary end points (maximum blood concentration after the first dose and the area under the concentration–time curve on the 3rd day), as measured by the ratio and 90% confidence interval (CI) of their geometric means: 92% (90% CI: 82–104%) and 106% (90% CI: 99–114%), respectively. The secondary PK parameters (daily maximum concentration at steady-state and time to maximum concentration) were also the same for the two formulations. The variability in all PK parameters was less for GTX-104 compared with oral nimodipine. The average oral bioavailability for nimodipine capsules was 7%.

### Rationale

GTX-104 approval will be sought through the 505(b)(2) regulatory pathway. The 505(b)(2) pathway is a way for drug sponsors to obtain Food and Drug Administration (FDA) approval for an already approved drug when at least some of the information about the safety and effectiveness of the drug comes from studies or findings not conducted by the sponsor and for which the sponsor does not have a right of reference or use. This pathway can be applicable to modified formulations or routes of administration of already approved drugs. It was determined by the sponsor after meetings with the FDA that such a pathway could be adequate for filing a new drug application for GTX-104 if the PK of GTX-104 was shown to approximate the PK of the approved product (oral nimodipine capsules). A single safety study for registration was thought to be adequate for approval by the 505(b)(2) pathway because a dose regimen of GTX-104 was developed that replicated the PK of oral nimodipine in human volunteers and because there is extensive safety and efficacy data for oral nimodipine over the last 40 years in thousands of patients [21, 22]. In the proposed study, we will use the same dose as the efficacious oral formulation, replicating plasma concentrations of every 4 h dosing of the oral formulation. The only difference is the route of administration.

Given the 505(b)(2) pathway, this protocol mimics the original pivotal oral nimodipine phase III clinical trials. Thus, the inclusion and exclusion criteria reflect a combination of considerations related to the oral nimodipine prescribing information and to general clinical trial design. We enroll the full spectrum of aSAH severity based on the Hunt and Hess scale because this scale was used in the original clinical trials, even though the clinical trials that were the basis for approval varied regarding grades included and doses used [23–26].

## Methods

### General Design

This safety study is a prospective, open-label, randomized (1:1 ratio), parallel group study of GTX-104 compared with oral nimodipine, in study participants with aSAH. Randomization is by central interactive response technology. Any health care professional authorized to be involved with the patient, the patient, and their authorized family members or surrogates and such can know what group the patient is assigned to. All study monitors and members of the sponsor can find out what group the patient is assigned to. The only people who are masked are the members of the end point adjudication committee (EAC) who assess the primary outcome. Approximately 100 study participants will be enrolled (approximately 50 study participants in each treatment group) at approximately 25 sites in the United States and Canada (Supplemental Table 1).

**Table 1:** Schedule of activities

Procedure	Screening ^a^	Treatment Phase			Follow-up Phase^d^	
Visit		Day 1	Day 2–20 ^c^	Day 21	Day 30	Day 90
Window					± 7 days	± 10 days
Informed consent	X					
Inclusion/exclusion criteria	X	X^b^				
Medical, surgical and psychiatric history	X					
Demographics	X					
Concomitant medications and procedures	X	X^b^	X	X	X	X
Physical and neurological exam^e^	X					
Vital signs (temperature and respiratory rate)^f^	X	X^b^	X	X		
Serum or urine pregnancy test	X				X	
Adverse events		X^b^	X	X	X	X
Clinical labs (hematology, chemistry, urine)^g^	X		X	X		
Blood pressure/heart rate^h^	X	X^b^	X	X		
12-lead electrocardiogram^i^	X	X	X	X		
C-SSRS (if capable)^j^	X			X	X	X
Computed tomography scan, angiography^k^	X					
Delayed cerebral ischemia, rescue therapy		X^b^	X	X		
Hunt and Hess and mGCS/WFNS	X	X^b^				
Randomization		X				
Investigational product administration^l^		X	X	X		
Therapeutic Intensity Scale^m^		X	X			
Health Outcomes^n^				X	X	X
Quality of Life (EQ-5D-3L)					X	X
Modified Rankin scale					X	X

Abbreviations: aSAH; aneurysmal subarachnoid hemorrhage, C-SSRS; Columbia-Suicide Severity Rating Scale, EQ-5D-3L; Euro QoL 5-dimension/3-level, mGCS; Modified Glasgow Coma Scale, WFNS; World Federation of Neurosurgical Societies.

^a^:Screening visit to occur before or after neurosurgical/endovascular repair of aneurysm. No study-specific assessments or procedures will be conducted prior to informed consent being obtained.

^b^:Baseline /Day 1 assessments to be performed prior to randomization. Screening assessments performed within 8 h prior to randomization, do not need to be repeated on Day 1, with the exception of the Hunt and Hess scale.

^c^:Patients who are discharged or discontinue the study prior to Day 21 will complete the Day 21 procedures.

^d^:Day 30 and Day 90 assessments to be performed ± 7 days and ± 10 days, respectively. If an in-person visit is not possible, the assessments can be performed remotely. Every effort should be made to collect adverse events, C-SSRS, modified Rankin scale and quality of life in all subjects.

^e^:Complete physical and neurological examination at screening. Targeted examinations as per standard of care.

^f^:Respiration rate and temperature to be recorded on electronic case report forms daily.

^g^:Clinical labs-Screening and Days 2, 7, 14 and 21 (± 1 day).

^h^:Blood pressure and heart rate are to be collected every hour on Days 1 to 3, every 4 h on Days 4 to 14 and every 12 h on Days 15 to 21. Blood pressure will be captured on the electronic case report forms if subjects demonstrate signs concerning for hypotension (e.g., dizziness, lightheadedness, excess somnolence).

^i^:12-lead electrocardiogram at Screening and Days 1, 7, 14 and 21 (± 1 day).

^j^:If a study participant has a score ≥ 4 or has a score that indicates active suicidal ideation or behavior on the C-SSRS, the subject’s mental health practitioner will be contacted immediately (if applicable), or the subject will be directed to the emergency department. Should a subject exhibit suicidal ideation or behavior while in hospital or have scores on the C-SSRS that reflect such thoughts, appropriate evaluation and treatment by a mental health professional while hospitalized should occur.

^k^:Computed tomography, magnetic resonance, or catheter angiography as per standard of care, aSAH history including repair procedure.

^l^:First dose of investigational product must be within 96 h of onset of aSAH.

^m^:Therapeutic Intensity Scale Day 1–14 only or until discharge from the intensive care unit, whichever comes first.

^n^:Health outcomes including number (calendar) of days in the intensive care unit, in hospital, on mechanical ventilation and discharge disposition should be completed upon hospital discharge.

### Objectives and End Points

The objectives are to evaluate the safety and tolerability and clinical and health economic outcomes of patients with aSAH treated with GTX-104 compared with those treated with oral nimodipine.

The primary end point is the incidence (percentage or proportion) of study participants with at least one episode of clinically significant hypotension with a reasonable possibility that GTX-104 or oral nimodipine caused the event, according to the masked EAC. The only common side effect of nimodipine is hypotension. That is why hypotension is the primary end point of the study. Hypotension is defined as a decrease in systolic BP > 20 mm Hg, diastolic BP > 10 mm Hg, or a systolic BP ≤ 100 mm Hg, confirmed by two consecutive readings within 5 min. Two categories of hypotension have been defined:

1. Not clinically significant: not requiring any medical treatment (pharmacotherapy or other intervention).
2. Clinically significant: requiring medical treatment, including but not limited to IV fluids, postural changes, dose reduction of investigational product (IP), interruption of antihypertensive medications, prescription of vasopressors, increasing dose of a vasopressor, or addition of a new vasopressor.

Secondary safety end points include the duration and total number of episodes of clinically significant hypotension (investigator opinion). This will be presented as number of study participants and number of events per study participant with descriptive statistics of the durations. The next secondary end point is the incidence and severity of adverse events. Reporting of adverse events follows good clinical practice guidelines and uses the incidence and severity of adverse events based on the National Cancer Institute Common Terminology Criteria for Adverse Events (version 5.0). Delayed cerebral ischemia (DCI) and rescue therapy are secondary end points. The definition of DCI is from Vergouwen et al. [27]. Rescue therapy may be administered in the event of angiographic vasospasm and/or DCI according to the site standard of care. Rescue therapy will be defined as induced hypertension, selective intraarterial infusion of vasodilator drugs, or balloon angioplasty. Information about angiographic vasospasm, DCI, and rescue therapy will be captured for this study. The protocol states that in general, off-label use of other drugs for the treatment of angiographic vasospasm and DCI, such as intraarterial nicardipine/verapamil, initiation of statin therapy, or infusion of magnesium for the purposes of achieving supratherapeutic blood magnesium concentrations are discouraged and should be avoided. This is consistent with guidelines [2]. It is recognized that some centers will use these drugs off-label for these indications.

Suicidal ideation is a secondary end point that will be assessed using the Columbia-Suicide Severity Rating Scale (C-SSRS) in all study participants who are able to provide the information. The C-SSRS will also be obtained at the end of the treatment period and at the day 30 and 90 follow-up visits. The C-SSRS is being assessed because the FDA issued a draft guidance in 2012 stating that “prospective suicidal ideation and behavior assessments should be carried out in all clinical trials involving any drug being developed for any psychiatric indication, as well as for all antiepileptic drugs and other neurologic drugs with central nervous system activity…” [28].

Clinical and health economic outcomes include hospital and intensive care unit lengths of stay, quality of life (EuroQol 5-dimension/3-level [EQ-5D-3L]) and modified Rankin Scale (mRS).

### Study Population

This reflects the pivotal oral nimodipine studies and the current indication for nimodipine [2, 14]. Oral nimodipine originally was approved for Hunt and Hess grades 1 to 3 [29]. This was expanded to all Hunt and Hess grades in 1996. This study therefore enrolls patients with aSAH with any Hunt and Hess grade at randomization. Although the primary clinical grading uses the Hunt and Hess scale, both the Hunt and Hess and World Federation of Neurological Surgeons grading scales are considered by the American Heart Association/American Stroke Association to be simple, validated scales for the initial assessment of the clinical severity of SAH [2, 29, 30]. The World Federation of Neurological Surgeons scale is widely used in the United States and has better interobserver and intraobserver reliability than the Hunt and Hess scale, so it also will be assessed before randomization [31].

Study participants will be male or female patients ≥ 18 years of age with a diagnosis of aSAH based on computed tomography and angiography (computed tomographic, magnetic resonance, or digital subtraction angiography). They must have a Hunt and Hess score from 1 to 5 just before randomization. Consent will be obtained for all patients from the patient or their guardian or legally authorized representative before any study procedures are done. The IP must be started within 96 h from the onset of the aSAH, as suggested in the prescribing information for oral nimodipine.

The exclusion criterion, which is consistent with the nimodipine prescribing information, is that patients receiving strong inhibitors of cytochrome P450 3A4 (CYP3A4) are excluded [21, 22].

Those who are excluded include patients with a history of recurrent syncope or hypotension, those requiring cardiopulmonary resuscitation within 4 days before randomization, and patients with second-degree or third-degree atrioventricular block or bradycardia (heart rate ≤ 50 bpm) before randomization. These are mentioned as warnings in the prescribing information and are exclusions because these may interfere with the safety assessments. Regulatory advice was to exclude patients with severely impaired liver function because they have impaired first-pass effect and decreased liver metabolism (cirrhosis [Child–Pugh class B and C] or alanine aminotransferase and/or aspartate aminotransferase more than 2.5 times the upper limit of normal). These patients can have high and unpredictable blood nimodipine concentrations leading to hypotension. Patients with a history of malabsorption syndrome, recent ileus (in the last 3 months) or other gastrointestinal conditions that would interfere with absorption of nimodipine, in the opinion of the investigator, are not eligible.

The protocol limits patients to 12 doses (or a total of 720 mg) of oral nimodipine (as a solution or capsules) as part of the standard of care for the ruptured aneurysm before randomization. Earlier versions of the protocol limited prestudy nimodipine to five doses (300 mg) because of concerns that longer times could limit the duration of exposure to GTX-104. This turned out not to be a problem, so the number of doses allowed was increased. Exclusion criteria based on general clinical trial principles include patients who are at imminent risk of death and/or have do not resuscitate orders and patients with a severe or unstable concomitant condition or disease other than what may be attributed to the aSAH that, in the opinion of the investigator, may increase the risk associated with study participation or nimodipine administration, or may interfere with the interpretation of study results.

### Study Procedures

**Fig. 1:**
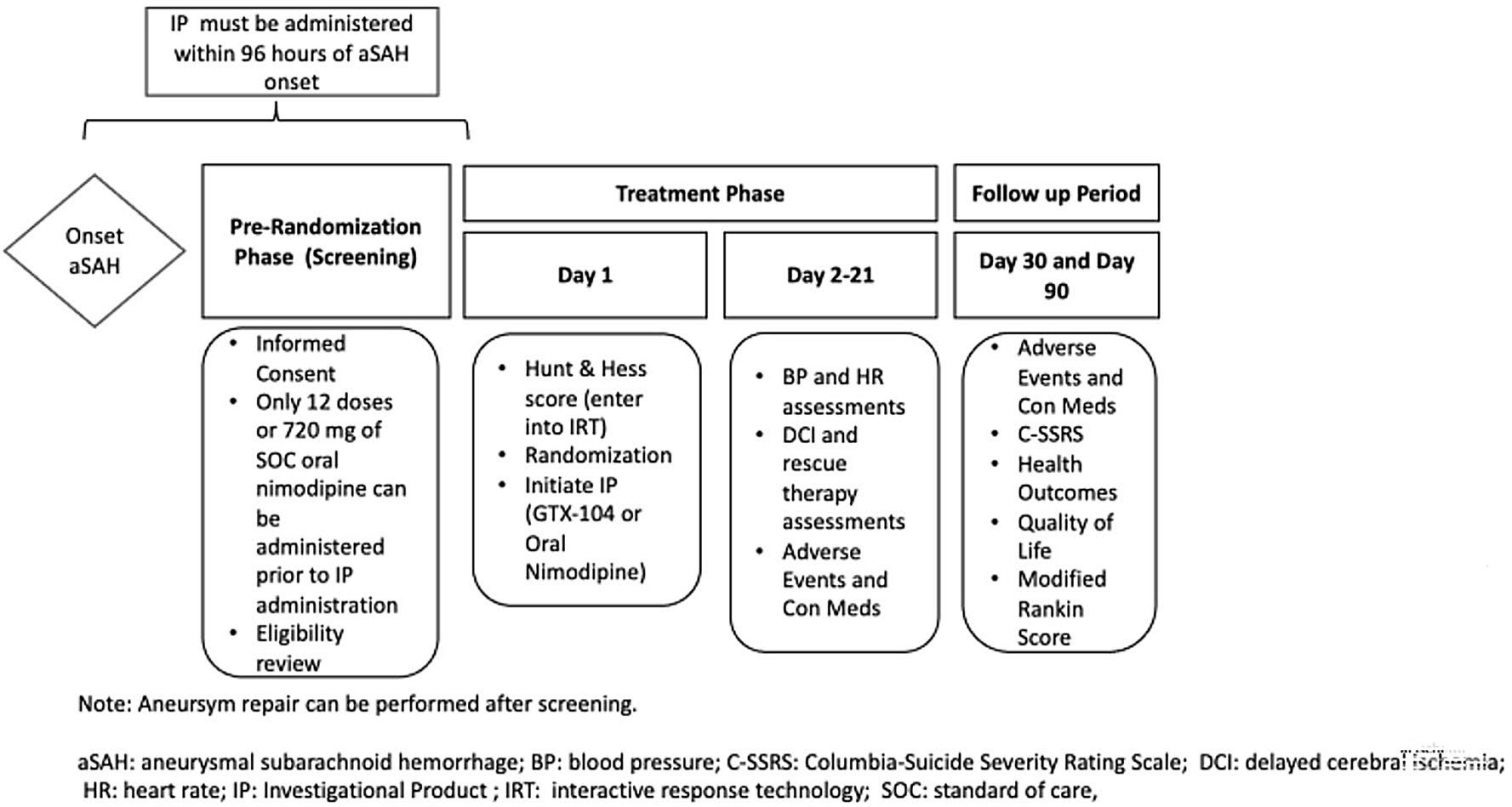
Flow chart of study protocol basics. aSAH, aneurysmal subarachnoid hemorrhage, BP, blood pressure, C-SSR, Columbia-Suicide Severity Rating Scale, DCI, delayed cerebral ischemia, HR, heart rate, IP, investigational product, IRT, interactive response technology, SOC standard of care

The study has prerandomization (screening), treatment and follow-up periods (Table 1, Fig. 1). Sites were selected based on many factors including number of aSAH cases per year, neurocritical care and neurovascular surgery infrastructure and experience, clinical trials infrastructure and experience, ability and willingness to comply with the study protocol, budget, and overall responsiveness or interest in the study. The study was approved by each site’s local or centralized ethics board. Regulatory and ethical aspects of the study adhere to consensus ethical principles derived from international guidelines including the Declaration of Helsinki and Council for International Organizations of Medical Sciences International Ethical Guidelines and applicable International Conference on Harmonization Good Clinical Practice Guidelines.

The study protocol does not require that the ruptured aneurysm be repaired. If the aneurysm is treated, which occurs in the vast majority of the patients who are eligible for this study, neurosurgical clipping or endovascular aneurysm occlusion are acceptable. The timing of aneurysm repair also is not specified for this study. Randomization will be in a 1:1 ratio to receive either GTX-104 or oral nimodipine for up to 21 days. Randomization will be stratified by Hunt and Hess score (1–3 vs. 4–5) and age (≤ 59 years vs. > 59 years). Additional data collected before randomization are what usually are documented in patients with aSAH (Table 1).

The treatment period begins at randomization (day 1) and continues up to day 21 or until the study participant is transferred to a location that cannot administer IP (e.g., discharge, regular hospital ward), whichever comes first. The focus of this period is documentation of adverse events and hypotension. Heart rate and BP will be measured every hour from day 1 to day 3, every 4 h from day 4 to day 14, and every 12 h from day 15 to day 21. In addition, BP will be captured if study participants demonstrate symptoms or signs or radiologic or laboratory findings concerning for hypotension (e.g., dizziness, lightheadedness, excess somnolence, electrocardiogram changes).

Follow-up continues until the day 90 assessments are completed. There also is a study visit at day 30. Specific outcomes measured in this period are the mRS, EQ-5D-3L) and health economic outcomes (days in the intensive care unit, in hospital and on mechanical ventilation, and discharge disposition) [32, 33].

#### IP

One arm of the study is administered GTX-104, which is a sterile solution containing nimodipine (2 mg/mL). The nimodipine is solubilized in polysorbate 80 micelles, 1.26% weight/volume of ultrapure alcohol, and sterile water. It is diluted in normal saline before administration. Polysorbate 80 is an excipient already used in other IV drugs, such as amiodarone and some chemotherapy drugs including docetaxel, epoetin/darepoetin and fosaprepitant [34, 35]. GTX-104 can be given through a peripheral or central venous catheter. All IV apparatus used cannot contain polyvinyl chloride. Nimodipine binds to polyvinyl chloride and polysorbate 80 can leach diethylhexyl phthalate from polyvinyl chloride [36].

The other arm of the study receives oral nimodipine, which is a gelatin capsule containing nimodipine, at 30 mg. Oral nimodipine is preferably administered in a fasting state (i.e., not less than 1 h before or 2 h after a meal). This is recommended in the prescribing information but anecdotally is not always adhered to in practice. If a study participant cannot swallow the nimodipine capsule, the instructions in the package insert are followed.

Study participants can receive liquid oral nimodipine solution before consent and randomization but not afterward. This is because this study is using a PK bridging strategy to gain FDA approval, and the dose of GTX-104 was selected to match the PK of the oral capsules, which are the reference standard from a regulatory perspective.

The management of study participants is left to the centers who are to use their standard of care.

If the site investigator deems it necessary, administration of IP can be interrupted, or the dose of IP can be reduced. The dose reduction should follow the recommendations in the FDA label [21]. The most recent label states that some patients, such as those taking moderate or weak CYP3A4 inhibitors, may require a nimodipine dose reduction in case of hypotension. The only specific statement about dose reduction pertains to those with severely disturbed liver function, in which the dosage may be reduced to 30 mg every 4 h. Furthermore, guidelines for management of aSAH state that the recommended dose should be given “even in the setting of nimodipine-induced hypotension that can be managed with standard medical interventions. However, if nimodipine causes significant BP variability, temporary stoppage may be necessary” [2].

Some investigators routinely administer oral nimodipine at 30 mg every 2 or 4 h. Administration every 2 h clearly does not follow the FDA label and therefore is not permitted in the protocol.

A principle of this study is to consider the dose regimens of the two drugs as if they are the same and to manage side effects the same way in each study group. For the conversion of doses between GTX-104 and oral nimodipine, 30 mg of oral nimodipine is equal to a 2-mg bolus of GTX-104.

### Concomitant Medications and Interventions

Concomitant administration of IP (either GTX-104 or oral nimodipine) and strong CYP3A4 inhibitors is contraindicated, based on the nimodipine prescribing information. Strong inhibitors of CYP3A4 include some macrolide antibiotics (e.g., clarithromycin, telithromycin), some anti-human immunodeficiency virus protease inhibitors (e.g., delaviridine, indinavir, nelfinavir, ritonavir, saquinavir), some azole antimycotics (e.g., ketoconazole, itraconazole, voriconazole) and some antidepressants (e.g., nefazadone). Grapefruit juice may potentiate the hypotensive effect of nimodipine. The BP lowering effect may last for 4 or more days after an intake of grapefruit juice, so drinking grapefruit juice or eating grapefruits is not recommended while taking nimodipine.

Strong CYP3A4 inducers (e.g., rifampin, phenobarbital, phenytoin, carbamazepine, St. John’s wort) should be avoided in patients prescribed nimodipine because they can lower the plasma concentration of nimodipine.

Selected concomitant medications and procedures will be recorded. These include medications with cardiovascular effects or that might affect absorption, metabolism, and excretion of nimodipine. Substantial procedures documented will include surgeries, ventricular catheter, lumbar puncture or drain, tracheostomy, gastrostomy, and neurointerventional procedures for angiographic vasospasm or DCI.

### End Point Adjudication, Data Management, and Data Monitoring Committees

An independent, masked EAC will review all clinically significant episodes of hypotension, regardless of suspected relatedness to IP. The committee consists of four neurocritical care physicians who are independent from the sponsor. They will be masked to the study participant’s study group. The EAC will decide if the hypotension is related to IP or not (yes or no). Two committee members will review each case and if they disagree, a third member will make the deciding assessment.

Independent contract research organizations (WuXi Clinical, Austin, TX; Anju, Phoenix, AZ; and Suvoda, Conshohocken, PA) are responsible for trial management. They conduct site recruitment and set up tasks, provide interactive response technology, site monitoring, manage electronic data capture, and security and drug safety.

A data monitoring committee will review the safety of GTX-104 and oral nimodipine when 25 and 50 study participants have completed the treatment period. They are three individuals (neurocritical care and neurosurgery) who are independent from the sponsor. A decision about subsequent reviews will be made after the first 50 patients are reviewed.

Before the start of the trial, the EAC and data monitoring committee had charters ratified that define the committee membership, roles, and responsibilities; meeting organization, format, and materials to be provided and reviewed; and meeting reports and recommendations.

### Statistics

The planned sample size is 100 study participants, with 50 in each arm. This sample size is not intended to provide adequate statistical power for specific comparisons or hypothesis tests of GTX-104 and oral nimodipine for any of the study end points. It is based on discussion with the FDA about how many patients would be adequate for a safety database of GTX-104 under the 505(b)(2) pathway. A sample size of 50 and an end point event with an incidence of 15% will provide an exact (Clopper-Pearson) 95% CI width of approximately 0.22, with a lower limit of 0.065, and an upper limit of 0.279. The 95% CI width at 50% incidence will be approximately 0.29.

The primary and all secondary end points will be presented based on a safety analysis set defined as all enrolled study participants according to their randomized group who receive at least one dose of IP after randomization. No missing data imputation will be applied. The two-sided 95% CI for the proportion will be obtained using the exact (Clopper-Pearson) method. The difference in the proportions between the two treatments and the exact 95% CI will also be presented.

Because the protocol allows GTX-104 patients to switch from IV to standard of care oral nimodipine, it is planned to analyze some study participants randomly assigned to GTX-104 based on whether they are receiving IP or standard of care oral nimodipine before the occurrence of the end point (modified safety analysis set). This is planned only for the secondary end points of duration and number of clinically significant hypotension events and the incidence and severity of adverse events. The other secondary end points and the clinical and health economic outcomes will only be summarized by the randomized treatment group because they are less likely to be affected by the change in formulation of nimodipine being administered.

If the day 90 quality of life and mRS are missing, the day 30 observation, if available, will be carried forward.

Duration of treatment with IP, total mass of standard of care oral nimodipine before randomization, total mass of IP during the treatment period and total mass of oral nimodipine after the switch from IV to oral during the treatment period, will be summarized. For each of the IP, the percent of the prescribed dose administered during the treatment period and a relative dose intensity will be calculated. The relative dose intensity will be defined as 100 × the total mass of IP administered/total mass of IP that should have been administered.

### Protocol Amendments

This protocol incorporates the 5th amendment. Supplemental Tables 2 to 6 summarize the changes for each amendment. The first version of the protocol was submitted to the FDA in April 2023. The first patient was enrolled in the study in September 2023. Protocol amendments 1 and 2 were submitted before this. This article was first submitted to *Neurocritical Care* in October 2024.

The key changes in amendment 1 were to simplify and clarify the primary and secondary end points that were initially based on a complicated and impractical hypotension assessment and were extremely poorly defined. Other changes harmonized the inclusion and exclusion criteria with the existing nimodipine prescribing information. Amendment 2 further simplified the primary end point to a clinically relevant easily assessable definition and added the mRS as a secondary end point. After the study started recruiting, amendment 4 allowed more time between hospital admission and randomization and clarified the definition of hypotension in response to questions from study sites. Amendment 5 also liberalized the definition of hypotension and brought the protocol recommendations for the management of oral nimodipine dose reductions in line with the prescribing information and the American Heart Association guidelines [2].

## Discussion

Intravenous nimodipine may have some advantages over oral nimodipine capsules. However, the safety and efficacy of nimodipine for aSAH relies heavily on one randomized clinical trial that used oral nimodipine tablets, at 60 mg every 4 h [26]. Gelatin capsules are used in the United States and tablets in Canada. The dosages of each are the same, but the bioavailability and peak plasma concentrations are higher with the gelatin capsules compared with the tablets [37]. The most important side effect of nimodipine is hypotension, which is closely associated with peak plasma concentrations greater than 30 to 40 ng/mL [12, 20, 38]. In healthy volunteers, this can be relatively easily controlled by adjusting the dose. However, plasma concentrations of nimodipine fluctuate more widely in patients with aSAH because of the variability in absorption and metabolism that occurs in these patients. The plasma concentration of oral nimodipine depends on the dose, dosage form, stomach contents, CYP3A4 activity in the small intestine wall and liver (first-pass effects), liver function, the patient’s cardiovascular condition, and what BP parameters are targeted for the patient. IV nimodipine, however, is not affected by most of these factors.

Food and first-pass effects are probably the most important sources of nimodipine plasma concentration variability. Bioavailability of oral nimodipine ranges from 3 to 28% in patients with aSAH and from 5 to 13% in healthy volunteers [37–41]. This suggests that by avoiding this variability, hypotension could be less common with IV nimodipine. Studies of GTX-104 in healthy human volunteers found less variability in PK with GTX-104 compared with oral nimodipine (unpublished data, Grace Therapeutics). Furthermore, gastrointestinal side effects such as diarrhea appear to be less common with IV nimodipine [5].

Additional rationale for the use of IV nimodipine is ease of use because it does not require patients to swallow the capsules or for the contents of capsules to be extracted for injection into gastric tubes. This could lead to improved compliance. There is no risk of inadvertent IV injection that is a black box warning with the capsules. GTX-104 also does not contain any preservatives or vessel-irritating components such as ethanol, so it can be injected through a peripheral venous catheter if desired. This also makes it theoretically better for intrathecal or intraventricular use than nicardipine, which contains preservatives, and for intraarterial use than the currently available IV nimodipine solution.

Hypotension is the primary end point of this study and the most important side effect of nimodipine. Hypotension is believed to be harmful to patients with aSAH especially from 3 to 14 days after the hemorrhage [2, 42]. When it occurs, the dosage of nimodipine often is reduced or nimodipine is stopped altogether. This is problematic because administration of the full recommended nimodipine dose has been associated with better outcome in some but not all reports [7, 43, 44]. Multivariate analysis of 220 patients with aSAH who were given oral nimodipine found only 96 (44%) got the full dosage for the first 14 days after aSAH. The dose was reduced to 50% in 63 (29%) patients and discontinued altogether in 61 (28%) patients. Multivariate analysis found unfavorable outcome was associated with increased age, worse Hunt Hess grade, and receiving less nimodipine [7]. Another study of 309 patients with aSAH found oral nimodipine dose was reduced or halted for at least 24 h between days 5 and 10 after SAH in 108 (53%) patients [44]. Patients who had dose reductions or stoppages were more likely to be poor grade and have higher Fisher scores on admission computed tomography. In multivariate analysis, the only factor associated with DCI was reduced nimodipine dose. Factors associated with poor outcome at 3 months follow-up were age, poor neurological grade, and reduced nimodipine dose. Other studies have associated DCI with reduced nimodipine dose [4, 19].

Most published data on IV nimodipine are from retrospective case series and prospective phase II studies [45–50]. There are two prospective randomized clinical trials comparing IV and oral nimodipine with placebo [51, 52]. Both prescribed IV nimodipine for 7 to 14 days followed by oral nimodipine. Jan, et al. [51] randomly assigned patients with aSAH within 24 h of neurological deterioration from vasospasm, so this was a treatment not a prophylaxis study. Nimodipine was associated with better clinical outcome, although the analysis was after excluding 61 of 188 randomly assigned patients. Most of the patients in Ohman et al. [52] were randomly assigned and started on nimodipine within 72 h of aSAH. There was no benefit of nimodipine on outcome at 3 months [52]. Thus, no conclusions can be drawn about the efficacy of continuous infusions of IV nimodipine.

Two randomized clinical trials compared oral and IV nimodipine and found no difference in clinical outcome, but the numbers of patients were too small to show noninferiority or differences in efficacy [53, 54]. No studies of IV nimodipine used the dose regimen that is given in this study. This dose regimen was chosen because it replicates the PK profile of oral nimodipine. Whether this profile is important for the efficacy of nimodipine is unknown, but this regimen fulfills the PK bridging usually required for this type of study.

## Supplementary Information

Below is the link to the electronic supplementary material.

Supplementary file1 (DOC 162 KB)
