# Supplementary material for: Description of STRIVE-ON Study Protocol: Safety and Tolerability of GTX-104 (Nimodipine Injection for IV Infusion) Compared with Oral Nimodipine in Patients Hospitalized for Aneurysmal Subarachnoid Hemorrhage (aSAH): A Prospective, Randomized, Phase III Trial (STRIVE-ON)
Source: Neurocrit Care. 2025 Jan 28;42(3):1107–17. doi: 10.1007/s12028-024-02207-8 (PMC12137520; doi:10.1007/s12028-024-02207-8)
Supplement: Supplementary file 1 — Supplementary file1 (DOC 162 KB) [file 12028_2024_2207_MOESM1_ESM.doc]

**Table 2:** Changes Incorporated into Protocol Amendment 1.0

| **Section of Protocol** | **Original Protocol** | **Change to Protocol and Rationale** |
| --- | --- | --- |
| **Objectives and Endpoints. Primary Endpoint** | Incidence (% or proportion of subjects) with grade ≥2 arterial hypotension possibly, probably, or definitely related to GTX-104/oral nimodipine. | Incidence (% or proportion of subjects) with at least 1 episode of grade ≥2 arterial hypotension with a reasonable possibility that GTX-104/oral nimodipine caused the event, according to the blinded endpoint adjudication committee (EAC).  The EAC assessment of hypotension will be the primary endpoint since the EAC will be blinded to the treatment assignment. This will help mitigate bias. The question/criteria to determine relationship to Investigational Product was clarified based on FDA Guidance “Safety Reporting Requirements for INDs and BA/BE Studies”, December 2012. |
| **Objectives and Endpoints. Secondary Endpoints** | Hemodynamic parameters (severity, duration and causality): systolic, diastolic and mean blood pressure and heart rate.  Grade ≥2 arterial hypotension (causality, duration, incidence [number of episodes, number (%) of subjects]).  Adverse Events.  Use of concomitant medications, rescue therapy and endovascular interventions for angiographic vasospasm and delayed cerebral ischemia.  Other vital sign assessments (body temperature and respiratory rate).  Suicidal ideation using the Columbia-Suicide Severity Rating Scale (C-SSRS).  Laboratory assessments (serum chemistry, hematology, urine analysis). | These were reordered, simplified and clarified to:  Duration and total number of episodes of grade ≥2 arterial hypotension.  Incidence and severity of Adverse Events (based on the National Cancer Institute Common Terminology Criteria for Adverse Events (NCI-CTCAE, Version 5.0).  Incidence of delayed cerebral ischemia and rescue therapy.  Suicidal ideation using the C-SSRS of ≥4.  The rationale is the hemodynamic parameters, other vital sign assessments and laboratory assessments are vague and not defined. These parameters/assessments are combined into the new secondary endpoint: Incidence and severity of Adverse Events based on the National Cancer Institute Common Terminology Criteria for Adverse Events (NCI-CTCAE, Version 5.0). This amendment clarifies that clinically significant changes to these parameters or assessments will be reported as Adverse Events.  The C-SSRS score will be dichotomized into <4 or ≥4 for each time it is assessed and then summarized by study arm because this cut-point in the scale defines those subjects who should be referred for immediate medical/psychiatric assessment. |
| **Exclusion Criteria** | Has received more than 3 doses of oral nimodipine (as a solution or capsules) as part of standard of care (SOC) for the ruptured aneurysm or within the previous 10 days before consideration for study entry and randomization into this clinical trial.  Is receiving cimetidine, or a strong inhibitor of CYP3A4, or a strong inducer of CYP3A4 Note: Use of cimetidine, or a strong inhibitor of CYP3A4 or a strong inducer of CYP3A4 prior to subarachnoid hemorrhage (SAH) and not within 5 times the drug’s half-life prior to randomization is allowed. | Has received more than 5 doses of oral nimodipine (as a solution or capsules) as part of SOC for the ruptured aneurysm prior to randomization.  The rationale is that allowing only 3 doses of oral nimodipine gives 16 hours of time. Since most sites start oral nimodipine as soon as aneurysmal SAH is diagnosed, this time would make it almost impossible to randomize any subjects into the study.  Is receiving strong inhibitors of CYP3A4 such as some macrolide antibiotics (e.g., clarithromycin, telithromycin), some anti-HIV protease inhibitors (e.g., delaviridine, indinavir, nelfinavir, ritonavir, saquinavir), some azole antimycotics (e.g, ketoconazole, itraconazole, voriconazole) and some antidepressants (e.g, nefazadone).  The rationale is exclusion criteria related to CYP3A4 inhibitors and inducers were changed to be consistent with the FDA label. |
| **Hypotension and Dose Reduction** | Clarified and added text for hypotension with regard to dose reduction of investigational product (IP). | Revised Hypotension and Dose Reduction section to write for this study, 3 grades of hypotension based on blood pressure (BP) and medical intervention have been defined as follows:  Decrease in systolic BP > 20 mm Hg or diastolic BP > 10 mm Hg, or a systolic BP ≤ 100 mm Hg, lasting for at least 5 minutes:   - Grade 1: not requiring any medical treatment (pharmacotherapy or other intervention). - Grade 2: requiring medical treatment without vasopressors, such as fluids, plasma or albumin perfusion, postural changes, dose reduction of IP or interruption of antihypertensive medications. - Grade 3: requiring prescription of vasopressors or increase in dose (by more than 10%) of vasopressor or addition of a new vasopressor.   Doses of IP can be modified, when necessary, based on individual subject tolerance to treatment. Every effort should be made to avoid dose reduction of IP. If required, dose reduction/discontinuation should proceed as follows:  For subjects experiencing Grade 1 hypotension, no alteration in dose is recommended.  For subjects experiencing Grade 2 hypotension:  Dose regimens should be reduced as follows: For GTX-104: 30 minute intravenous (IV) bolus reduced to 2 mg every 4 hours (the background continuous IV infusion remains 0.15 mg/hour). For oral nimodipine: reduced to one 30 mg capsule every 4 hours. When at least 8 hours have elapsed since the hypotension resolves, the dose of IP may be increased to the full recommended dose, based on a medically-qualified investigator’s decision.  For subjects experiencing Grade 3 hypotension: Treatment should be temporarily stopped. When at least 8 hours have elapsed since the hypotension resolves and there have not been escalating vasopressor requirements, dosing may be resumed at 50% of the initial dose, based on a medically-qualified investigator’s decision: For GTX-104: 30 minute IV bolus of 2 mg every 4 hours (the background continuous IV infusion remains 0.15 mg/hour). For oral nimodipine: one 30 mg capsule every 4 hours. After at least 8 hours of tolerating the above regimen, the dosing of IP may be increased to 100% of the initial dose, based on a medically qualified investigator’s decision. For GTX-104: continuous IV infusion of 0.15 mg/hour and a 30 minute IV bolus of 4 mg every 4 hours. For oral nimodipine: two 30 mg capsules every 4 hours.  Note 1 was deleted. It wrote that by definition, subjects with grade 3 hypotension receive vasopressor(s). Therefore, resolution to grade 1, baseline, or to within normal range is considered after 12 hours of stable or reduced dose of vasopressors.  The rationale is that definitions of hypotension were added to this section and the instructions for dose reduction/stopping were clarified. The hypotension definitions are called grades 1 to 3 but these are not the grades used for assessing severity of adverse events. |
| **Concomitant Medications and Procedures** | Updated requirements regarding concomitant medications and interventions to harmonize with FDA label. | As written above, concomitant administration of IP (either GTX-104 or oral nimodipine) and strong CYP3A4 inhibitors is contraindicated. Strong inhibitors of CYP3A4 include some macrolide antibiotics (e.g., clarithromycin, telithromycin), some anti-HIV protease inhibitors (e.g., delaviridine, indinavir, nelfinavir, ritonavir, saquinavir), some azole antimycotics (e.g., ketoconazole, itraconazole, voriconazole) and some antidepressants (e.g., nefazadone).  After intake of grapefruit juice and nimodipine, the blood pressure lowering effect may last for at least 4 days after the last ingestion of grapefruit juice. Ingestion of grapefruit / grapefruit juice is therefore not recommended while taking nimodipine.  If nimodipine is concomitantly administered with moderate and weak inhibitors of CYP3A4, BP should be monitored and if hypotension occurs, a reduction of the IP dose may be necessary.  Concomitant administration of other calcium channel blockers should be avoided, titrated down, or limited to the first few hours or days of nimodipine therapy. Concomitant administration of other anti-hypertensive agents may require dose adjustment of such agents.  Concomitant interventions are common in subjects with SAH, including but not limited to intensive care or hospital (re)admission, ventricular drainage, lumbar puncture, mechanical ventilation, use of nasogastric or gastric tubes and tracheostomy. Use of concomitant interventions will be captured in the electronic case report form.  The rationale is the contraindications for the strong CYP3A4 inhibitors and the intake of grapefruit juice are now consistent with the approved product information. |
| **Data Monitoring Committee** | Added a separate Data Monitoring Committee (DMC). | An independent, blinded Endpoint Adjudication Committee (EAC) will review all episodes of hypotension, regardless of suspected relatedness to IP.  The DMC will review the safety of GTX-104 and oral nimodipine according to a pre-specified schedule, either on a calendar basis or based on the number of subjects treated.  Members of the EAC and DMC will each consist of 3 to 5 independent experts with relevant medical and scientific experience. Prior to the start of the trial, the EAC and DMC will have charters ratified. |

**Table 3:** Changes Incorporated into Protocol Amendment 2

| **Section of Protocol** | **Existing Protocol** | **Change to Protocol and Rationale** |
| --- | --- | --- |
| **Primary and Secondary endpoints** | Incidence (% or proportion of subjects) with at least 1 episode of grade ≥2 arterial hypotension with a reasonable possibility that GTX-104/oral nimodipine caused the event, according to the blinded Endpoint Adjudication Committee (EAC). | Incidence (% or proportion of subjects) with at least one episode of clinically significant hypotension with a reasonable possibility that GTX-104/oral nimodipine caused the event, according to the blinded EAC.  The grades and definitions of hypotension were changed.  The rationale is that the grading system was complicated and not clinically relevant. Essentially the only important hypotension is that which the investigator does something about, which is now defined as clinically significant hypotension. The primary endpoint has been updated to incidence (% or proportion) of subjects with at least 1 episode of clinically significant hypotension. |
| **Clinical and Health Economic Outcomes** |  | Added modified Rankin Scale (mRS) to be assessed at Day 30 and Day 90.  The rationale is that the mRS is the most common functional outcome scale used to assess outcomes after stroke, including after aneurysmal subarachnoid hemorrhage (aSAH). It has been added as a general assessment at up to 3 months after the aSAH of the effects of any adverse events that occur. |
| **Hypotension and Dose Reduction** | This is described in Table 2 under the **Hypotension and Dose Reduction** section 3^rd^ column (**Change to Protocol and Rationale**) | For this study, hypotension is defined as follows:  Decrease in systolic blood pressure (BP) > 20 mm Hg or diastolic BP > 10 mm Hg, or a systolic BP ≤ 100 mm Hg, confirmed by 2 consecutive readings within 5 minutes.  The rationale is to clarify how BP readings should be confirmed and for consistency with other sections of the protocol.  Two categories of hypotension have been defined as follows:  1. Not clinically significant: not requiring any medical treatment (pharmacotherapy or other intervention).  2. Clinically significant: requiring medical treatment, including but not limited to intravenous fluids, postural changes, dose reduction of IP, interruption of antihypertensive medications, prescription of vasopressors, increasing dose of a vasopressor or addition of a new vasopressor.  Hypotension definitions were collapsed to 2 categories that are consistent with the primary endpoint: not clinically significant (previously grade 1, defined as not treated) and clinically significant (previously grade 2 or 3, now collapsed to one category since they are both hypotension events that are treated). The terminology was changed from “grade” to "not clinically significant/ clinically significant" to avoid confusion with severity grading used for adverse events.  The dose reduction guidelines have been modified to align with the two categories of hypotension and to delete the option to stop/restart the IP. Stopping IP is always an option in any clinical trial and doesn’t need to be specified. The section now reads:  Since nimodipine is administered as standard of care (SOC) to patients with aSAH, every effort should be made to give the entire prescribed dose of investigational product (IP) and to avoid dose reductions or interruptions of IP.  If deemed necessary based on investigator decision, dose reduction of IP should proceed according to SOC. The recommended dose reductions may be: For GTX-104: 30 minute IV bolus of 2 mg every 4 hours. The background of continuous infusion remains 0.15 mg/hr. For oral nimodipine: One 30 mg capsule every 4 hours.  The rationale to recommend these dose reductions was based on the fact that this is basically SOC at many centers even though this is not recommended in the prescribing information for oral nimodipine capsules. |
| **Suicidal Ideation and Behavior Risk Monitoring** | Suicidal Ideation  and Behavior Risk  Monitoring | The Columbia-Suicide Severity Rating Scale (C-SSRS) Initial or Baseline Form will also be completed at screening (if subject is capable) or the first time the assessment is completed.  The rationale is that this is consistent with how the C-SSRS is used in clinical practice. The baseline form should be  completed the first time the scale is performed so that the lifetime scale is obtained.  The protocol also added that if a subject has a score ≥4 on the C-SSRS or evidence of suicidal behavior is noted, the subject should be referred to a mental health professional for evaluation and potential treatment.  The rationale is standard, proper medical care and patient safety for any subject with active suicidal ideation. If a subject has a score ≥4 on the C-SSRS or evidence of suicidal behavior is noted, the subject should be referred to a mental health professional for evaluation and potential treatment. |

**Table 4:** Changes Incorporated into Protocol Amendment 3

| **Columbia-Suicide Severity Rating Scale (C-SSRS)** | If a subject has a score ≥4 on the C-SSRS, or evidence of suicidal behavior is noted, the subject should be referred to a mental health professional for evaluation and potential treatment. | If a subject has a score ≥4 or has a score that indicates active suicidal ideation or behavior, on the C-SSRS, the subject’s mental health practitioner will be contacted immediately (if applicable) or the subject will be directed to the emergency department. Should a subject exhibit suicidal ideation or behavior while in hospital or have scores on the C-SSRS that reflect such thoughts, appropriate evaluation and treatment by a mental health professional while hospitalized should occur.  The rationale is to bring the management of patients with a score ≥4 into agreement with best medical practice. |
| --- | --- | --- |
| **Blood Pressure (BP) and Heart Rate** | Not mentioned. | Added to the protocol was that BP will be captured in the electronic case report forms when subjects demonstrate signs concerning for hypotension (e.g. dizziness, lightheadedness, excess somnolence).  The rationale was that the FDA was concerned we would miss symptomatic hypotension episodes. |

**Table 5:** Changes Incorporated into Protocol Amendment 4

| **Section of Protocol** | **Existing Protocol** | **Change to Protocol and Rationale** |
| --- | --- | --- |
| **Exclusion criteria** | Has received more than 5 doses of oral nimodipine (as a solution or capsules) as part of the standard of care (SOC) for the ruptured aneurysm prior to randomization. | Changed this exclusion to has received more than 12 doses (or 720 mg) of oral nimodipine (as a solution or capsules) as part of the SOC for the ruptured aneurysm prior to randomization.  The limitation to 5 doses in the previous protocol version was to maximize patient exposure to GTX-104. At the time of this amendment proposal, all patients receiving GTX-104 received the infusion for a minimum of 7 days and a maximum of 21 days. This exposure was similar to the nimodipine exposure in the oral nimodipine arm of the study.  Therefore, this amendment proposed to increase the allowable oral nimodipine SOC prior to randomization to 12 doses (or 720 mg). The rationale was to allow more time for screening and randomization of subjects. |
| **Hypotension and Dose Reductions** |  | Added to the criteria for hypotension that fluctuations in blood pressure (BP) are common in patients with aneurysmal subarachnoid hemorrhage (aSAH). Sites should follow their SOC procedures when assessing and treating changes in BP. The protocol does not necessarily require repeat measurements within 5 minutes even if the BP itself decreases enough to meet the definition (other than the repeat within 5 minutes) above.  The rationale as to clarify and provide guidance for assessing changes in BP. If the BP decreases that decrease may be medically fine and not of concern and this is meant to clarify that the protocol doesn’t require the BP to be measured again in such situations. |

**Table 6:** Changes Incorporated into Protocol Amendment 5

| **Section** | **Description of Change** | **Summary of Changes and Rationale** |
| --- | --- | --- |
| **Hypotension and Dose Reduction** | Changes to blood pressure (BP) evaluation and dose reductions. | A statement was added to clarify evaluation of not clinically significant hypotension events: Fluctuations in BP are common in patients with aneurysmal subarachnoid hemorrhage (aSAH). Sites should follow their standard of care (SOC) when assessing and treating changes in BP. If BP drops and meets the definition of hypotension above, is repeated within 5 minutes and is not treated, these events should be reported as not clinically significant. In the event that the BP measurement is not repeated within 5 minutes yet medical treatment is rendered, the event should be considered clinically significant.  The rationale is that since the SOC at most sites is to repeat measurement of BP every 15 minutes in the first hours to days after aSAH, text has been added to address the situation in which there has not been two measurements of BP within 5 minutes. In this situation if the first reading results in treatment of hypotension, the sites should record the event as clinically significant and the event should be adjudicated by the Endpoint Adjudication Committee.  The dose reduction recommendations also were revised again. The protocol now states that if deemed necessary based on investigator decision, dose reduction should proceed according to the US label (Nimodipine Package Insert). The recommended dose reductions may be: For GTX-104: 30 minute intravenous (IV) bolus of 2 mg every 4 hours. The background continuous IV infusion remains 0.15 mg/hour. For oral nimodipine: One 30 mg capsule every 4 hours. Information about dose reduction and discontinuation will be captured in the electronic case report forms.  The rationale to remove the recommended dose reductions to dosing every 2 hours (30 minute IV bolus of 2 mg every 2 hours or one 30 mg capsule every 2 hours) was based on FDA recommendations that the dose regimen follow the US label for oral nimodipine which does not mention dosing every 2 hours. |
